# Impact of Prostate Size on the Outcomes of Radical Prostatectomy: A Systematic Review and Meta-Analysis

**DOI:** 10.3390/cancers13236130

**Published:** 2021-12-05

**Authors:** Omar Fahmy, Nabil A. Alhakamy, Osama A. A. Ahmed, Mohd Ghani Khairul-Asri

**Affiliations:** 1 Department of Urology, Universiti Putra Malaysia (UPM), Serdang 43400, Malaysia; omarfahmy.ahmed@upm.edu.my; 2 Department of Pharmaceutics & Industrial Pharmacy, Faculty of Pharmacy, King Abdulaziz University, Jeddah 21589, Saudi Arabia; nalhakamy@kau.edu.sa (N.A.A.); oaahmed@kau.edu.sa (O.A.A.A.)

**Keywords:** prostate size, prostate volume, prostate weight, radical prostatectomy, prostate cancer

## Abstract

**Simple Summary:**

Prostate size can vary widely among men regardless of whether they have prostate cancer or not. Many studies reported very conflicting results regarding the impact of prostate size on the outcome of radical prostatectomy. This is the first systematic review and meta-analysis on this topic to investigate the impact of prostate size on the operative, functional and oncological outcomes of radical prostatectomy. In general, a smaller prostate can be associated with fewer surgical complications, but with a higher chance of positive surgical margins. This can be useful when counseling patients before surgery.

**Abstract:**

Background: The impact of prostate size on the radical prostatectomy outcome is not clear. Several published reports have shown conflicting results. Objectives: To investigate the effect of prostate size on the surgical, functional and oncological results of radical prostatectomy. Methods: A systematic review and meta-analysis were carried out in accordance with the PRISMA criteria. Finally, we investigated the research that reported on the impact of prostate size on radical prostatectomy outcome. The Review Manager (RevMan) software version 5.4 was utilized for statistical analysis. Results: Eighteen studies including 12,242 patients were included. Estimated blood loss was significantly less with smaller prostates (Z = 3.01; *p* = 0.003). The complications rate was 17% with larger prostates, compared to 10% for smaller prostates (Z = 5.73; *p* < 0.00001). Seventy-three percent of patients with a smaller prostate were continent within one month, compared to 64% with a larger prostate (Z = 1.59; *p* = 0.11). The rate of positive surgical margins was significantly higher with smaller prostates (20.2% vs. 17.8%). (Z = 2.52; *p* = 0.01). The incidence of biochemical recurrence was higher with smaller prostates (7.8% vs. 4.9%) (Z = 1.87; *p* = 0.06). Conclusion: Larger prostate size is associated with more blood loss and a higher rate of complications. However, the oncological outcome is better, compared to that in patients with smaller prostates. The impact of the size on the functional outcome is not clear.

## 1. Introduction

Prostate cancer is the second most prevalent cancer in men, and the fifth leading cause of death worldwide. In 2018, about 1.2 million new cases of prostate cancer were reported globally, with a higher incidence noticed in developed nations [1]. Radical prostatectomy (RP) is one of the main treatment options for prostate cancer including locally advanced disease, with roughly half of all prostate cancer patients undergoing this operation [2].

RP entails removing the whole prostate between the urethra and the bladder, including the seminal vesicles and the surrounding fascial tissue, to provide a negative surgical margin. Terence Millin, an Irish surgeon, firstly introduced this procedure in 1945 [3], and then it was improved in 1982 by Patrick C. Walsh, who introduced the contemporary nerve-sparing, retropubic RP to maintain patients’ potency [4]. William Schuessler et al. performed the first laparoscopic RP (LRP) in 1991 to reduce the morbidity of open surgery and overcome the challenging exposure of the retropubic region during open RP [5]. In 2000, Binder et al. executed the first robot-assisted RP (RARP) in Frankfurt, Germany [6], and currently more than 80% of RP are performed using robotic platforms [7].

It is well-known that prostate size varies widely between men and might reach up to several folds of the normal size. This variability in size can be associated with other anatomical variations since the prostate is located in a narrow space and surrounded by many structures. Several studies reported on the impact of prostate size on the surgical, functional, and oncological outcomes of RP, but the results are very conflicting, and no consensus has been reached thus far [8,9,10,11].

To the best of our knowledge, this work is the first systematic review and meta-analysis to investigate the impact of prostate volume on RP outcomes, aiming to resolve the long-standing debate regarding this topic.

## 2. Materials and Methods

### 2.1. Search Strategy

According to PRIMSA criteria [12], an online systematic search was conducted through online data bases (PubMed, EMBASE, Wiley Online Library and Cochrane databases). The following keywords were utilized: prostate cancer; radical prostatectomy; prostate volume; prostate size, laparoscopic prostatectomy; robotic prostatectomy. Exclusion criteria were: (1) review articles, (2) case reports, (3) letters to editors and editorial comments, (4) repeated publications for the same author, or from the same center, (5) studies with no data on the impact of prostate size on the outcome, and (6) non-English articles. All initial results underwent title or abstract assessment, followed by full-text assessment for the selected publications. Finally, all studies reporting on the impact of prostate size on the outcome with data eligible for pooled analysis were included. Our study has been registered on the INPLASY platform under number 2021110035.

### 2.2. Data Extraction

Data were independently extracted by two authors and checked by a third one, and included total number of patients, time frame, method of prostate volume assessment (specimen, MRI, or transrectal ultrasound), and surgical technique (open, laparoscopic, or robotic). Continuous data included operative time (OT), console time (CT), estimated blood loss (EBL), and mean and standard deviation (SD). When data were reported as median, range, or interquartile range, Wan’s equation was applied to estimate the mean and SD from median, range/interquartile range, and sample size [13]. For dichotomous data, such as complications, blood transfusion (BT), number of continent patients, positive surgical margins (PSM), number of patients that developed biochemical recurrence (BCR), the numbers of events, and total number of patients were extracted. Odds ratios (OR) with 95% confidence intervals (CI) for the impact of prostate volume on the outcome were extracted when reported.

### 2.3. Primary Outcomes

The primary outcome of this systematic review and meta-analysis was to investigate the impact of prostate volume on the perioperative, oncological, and functional outcomes of RP. Based on the extracted data, perioperative outcomes were assessed by OT, CT, EBL, BT, complications, and bladder neck stenosis (BNS). Functional outcome was represented by continence recovery. Early continence was defined as achieving continence within 1 month post-operatively. Late continence was defined as achieving continence 6 to 12 months postoperatively. Impact of prostate volume on oncological outcome was investigated by PSM and BCR.

### 2.4. Statistical Analysis

The Nordic Cochrane Centre, The Cochrane Collaboration, Copenhagen, employed Review Manager (RevMan) software version 5.4 for statistical analysis and the creation of forest plots for this meta-analysis. The mean difference with 95% CI was utilized for comparing continuous data (EBL, OT, CT). For dichotomous data, we utilized the odds ratio (OR) with 95% CI. Pooled OR was calculated from number of events and total sample size or using Log OR and standard of error (SE) if OR and 95%CI were reported. When pooled analysis for OR could be performed by the two modalities for the same outcome, both modalities were used such as in continence and BCR. Random mode was employed in all the analyses regardless of the value of I^2^ to minimize the effect of heterogenicity of the studies on the outcome. The Z-test was used to assess the overall impact. *p*-values < 0.05 were deemed significant in all tests.

## 3. Results

### 3.1. Search Results

**Figure 1:**
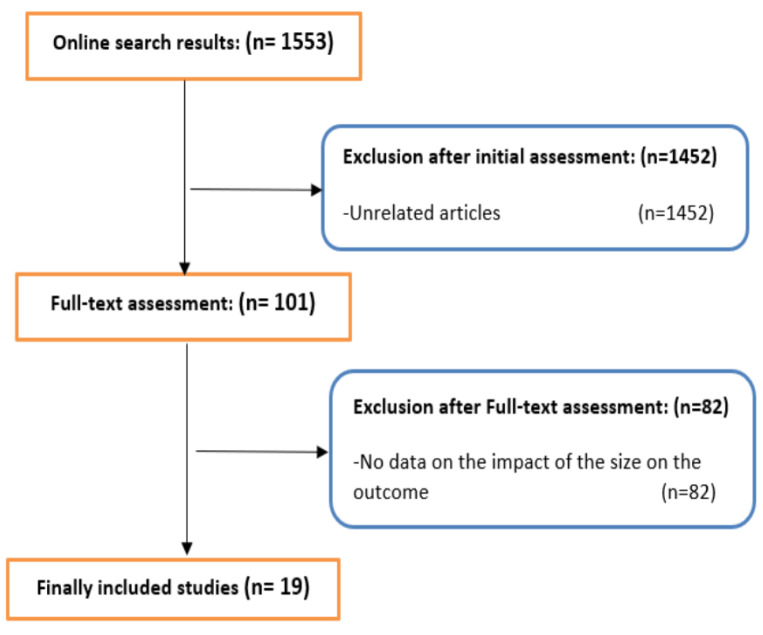
CONSORT diagram demonstrates the selection process for the included studies.

Following the process demonstrated in the CONSORT diagram in Figure 1, 19 publications including a total of 13,844 patients were eventually involved [8,9,10,11,14,15,16,17,18,19,20,21,22,23,24,25,26,27]. Totals of 6163 (44.5%), 5557 (40.1%), and 2124 (15.3%) patients underwent RARP, LRP, and open RP, respectively. The included studies are summarized in Table 1.

### 3.2. Perioperative Outcomes

**Figure 2:**
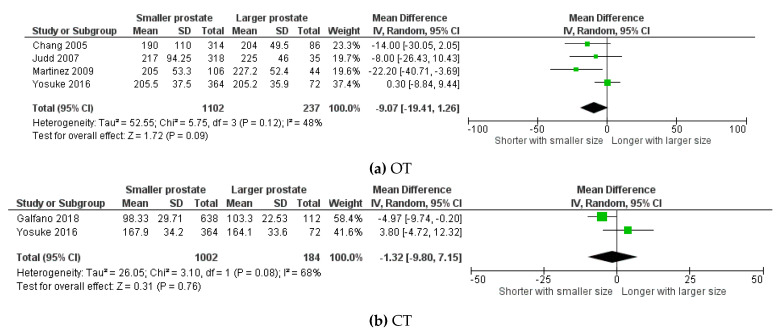
Forest plots for the comparison of (**a**) OT: operative time, (**b**) CT: console time.

Four studies including 1339 (1102 vs. 237) patients were included in the comparison of OT [9,10,16,26]. Overall, OT for patients with smaller prostates was shorter with a *p* value showing a trend towards significance; however, it did not reach the significance level (Z = 1.72; *p* = 0.09; 95%CI −19.41:1.26) (Figure 2a). For CT, two studies including 1186 (1002 vs. 184) patients were included [11,26]. No significant difference was noticed. (Z = 0.31; *p* = 0.76; 95%CI −9.80: 7.15) (Figure 2b).

**Figure 3:**
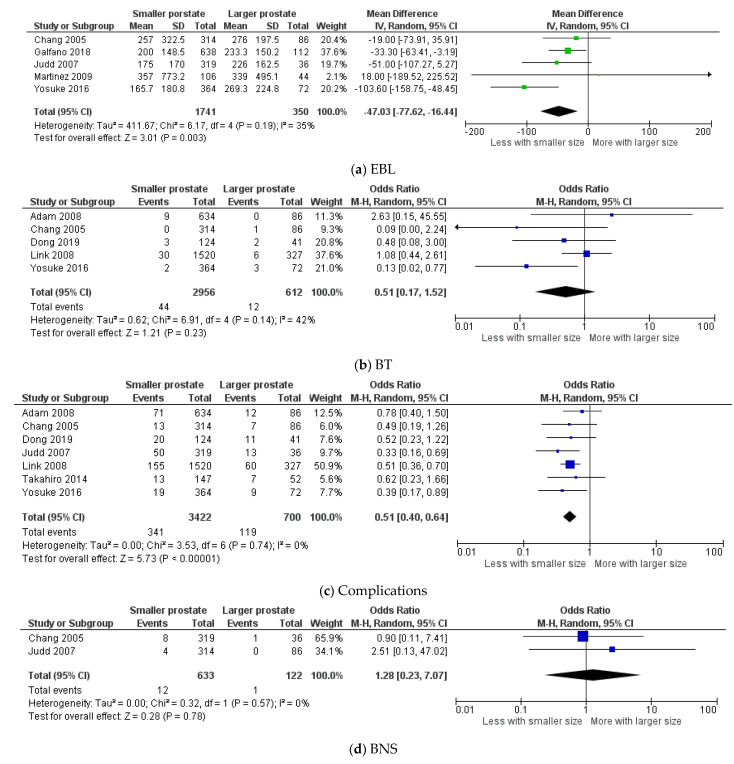
Forest plots for the comparison of (**a**) EBL: estimated blood loss, (**b**) BT: blood transfusion, (**c**) complications, (**d**) BNS: bladder neck stenosis.

Five studies with 2091 (1741 vs. 350) patients were included in the comparison of EBL [9,10,11,16,26]. EBL was significantly less in patients with smaller prostates (Z = 3.01; *p* = 0.003; 95%CI −77.62: −16.44) (Figure 3a). However, there was no significant difference in BT; the overall transfusion rate was 1.5% (44/2956) for patients with smaller prostates compared to 2% (12/612) for those with larger prostates (Z = 1.21; *p* = 0.23; 95%CI 0.17: 1.52) (Figure 3b) [8,10,24,26,27].

Overall, complications were significantly higher in patients with larger prostates. From seven studies containing 4122 patients [8,9,10,20,24,26,27], the incidence of complications was 17% (119/700) in patients with larger prostates, compared to 10% (341/3422) for those with smaller prostates (Z = 5.73; *p* < 0.00001; 95%CI 0.40: 0.64) (Figure 3c). A separate analysis for BNS was feasible from two studies containing 755 patients, and the incidence was 1.9% (12/633) vs. 0.8% (1/122) for patients with smaller and larger prostates, respectively, yet the difference was insignificant (Z = 0.28; *p* = 0.78; 95%CI 0.23: 7.07) [9,10] (Figure 3d).

### 3.3. Functional Outcomes

**Figure 4:**
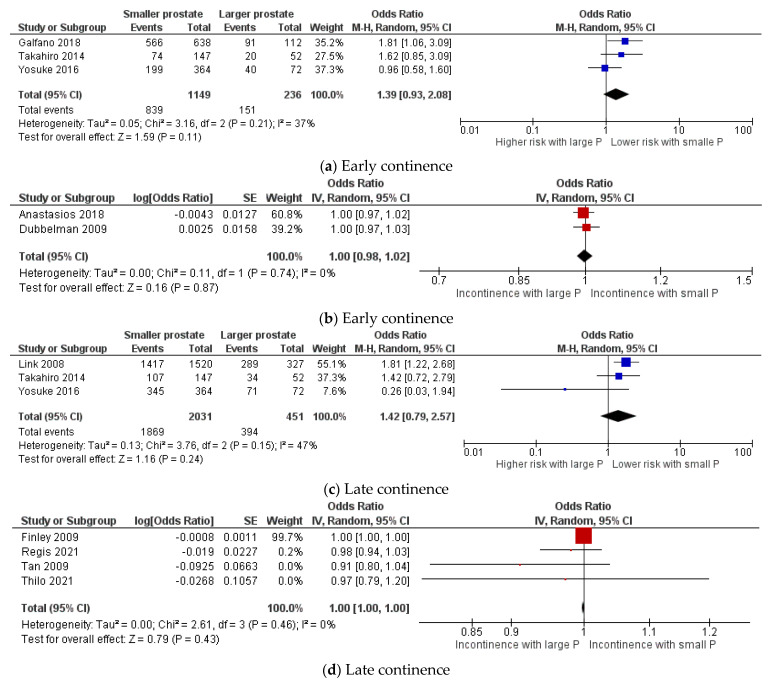
Forest plots for comparison of early continence (**a**,**b**) and late continence (**c**,**d**). P: prostate.

Patients with smaller prostates showed a trend toward better early continence; however, it was insignificant. From three studies including 1385 patients, 73% (839/1149) were continent within 1 month, compared to 64% (151/236) with larger prostates (Z = 1.59; *p* = 0.11; 95%CI 0.93: 2.08) [11,20,26] (Figure 4a). Two studies with 149 patients that reported OR for early continence were included in a pooled analysis that displayed no effect for prostate volume on early continence (Z = 0.16; *p* = 0.87; 95%CI 0.98: 1.02) (Figure 4b) [14,15].

For late continence (6 to 12 months after surgery), there was no significant difference. In three studies containing 2482 patients, 92% (1869/2031) with smaller prostates were continent compared to 87% (394/451) with larger prostates (Z = 1.16; *p* = 0.24; 95%CI 0.979: 2.57) [20,24,26] (Figure 4c). Four studies including 2984 patients that reported OR for late continence were included in a pooled analysis that displayed no difference between patients with smaller and larger prostates (Z = 0.79; *p* = 0.43; 95%CI 1.00: 1.00) (Figure 4d) [17,21,22,25].

### 3.4. Oncological Outcomes

**Figure 5:**
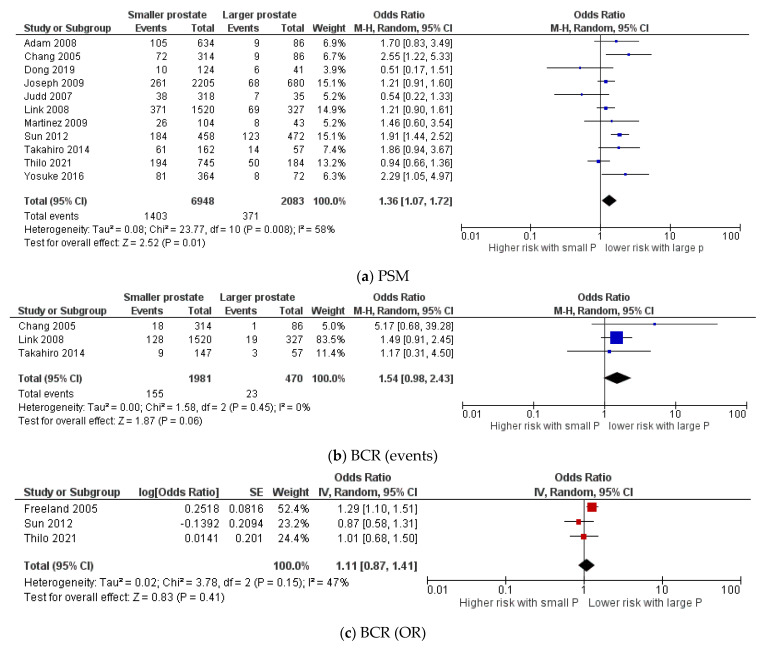
Forest plots for comparison of (**a**) PSM: positive surgical margins, (**b**,**c**) BCR: biochemical recurrence. P: prostate.

Surprisingly, the rate of PSM was significantly higher in patients with smaller prostates. From 11 studies with a total of 9031 patients, 20.2% (1403/6948) had PSM, compared to 17.8% (371/2083) of patients with larger prostates. (Z = 2.52; *p* = 0.01; 95%CI 1.07: 1.72) [8,9,10,16,19,20,21,23,24,26,27] (Figure 5a). In correlation with PSM, the number of patients that developed BCR was higher in those with smaller prostates, with a *p* value almost significant. In 2451 patients from three studies, 7.8% (155/1981) developed BCR, compared to 4.9% (23/470) with larger prostates (Z = 1.87; *p* = 0.06; 95%CI 0.98: 2.43) [10,20,24] (Figure 5b). Pooled analysis of Log OR and SE of three studies including 3361 patients displayed insignificant higher risk of BCR in patients with smaller prostates (Z = 0.83; *p* = 0.41; 95%CI 0.87: 1.41) [19,21,28] (Figure 5c).

## 4. Discussion

Currently, RP is one of the principal choices for treatment of prostate cancer; however, its functional outcomes discourage some patients from accepting this treatment modality [29]. Over many years, RP has undergone significant development in terms of the approach and utilization of minimally invasive surgery to improve the outcome. In addition, factors that affect the outcome have been extensively investigated [30]. Prostate size is one of the anatomical factors that can vary widely among patients. Unlike some treatment modalities, such as brachytherapy, prostate volume is not a contraindication for RP [31]. However, thus far the impact of prostate volume on RP outcome remains unclear. Several studies have reported on the impact of prostate size on RP outcome, but there is marked controversy among the published results [23,24,26,27]. This comprehensive review sought to resolve the current conflict and address the real impact of prostate volume on surgical, functional, and oncological outcomes of RP.

The prostatic gland is an organ ovoid in shape, located in the retropubic space and underneath the urinary bladder, and is traversed by the prostatic urethra, the junction between the bladder neck and membranous urethra. It has anterior, posterior, and lateral surfaces with the prostatic base facing the bladder neck upwards, while the apex faces downwards very close to the external urinary sphincter. In addition, the prostate is surrounded by fascial layers where the neurovascular bundles run [32]. Theoretically, the increase in prostate size in this limited space will add more challenges and difficulty to surgical removal of the prostate. Furthermore, it can be associated with anatomical changes, such as widening of the bladder neck, protrusion of the median lobe of the prostate inside the urinary bladder, accessory blood supply, and elongation of the prostatic urethra, and also the subsequent gap between the bladder neck and the urethral stump after removal of the prostate.

The normal size of the prostate is about 20 cm^3^; however, it can reach up to a few hundred cm^3^ [33]. Until now, there has been no definition for a large prostate, and usually subjective terms have been used to describe an enlarged prostate, such as, mild, moderate, or huge. The studies included in this meta-analysis used different cut-off volume values to assess the impact of the size on RP outcome. The reported cut-off varied between 40 and 100 cm^3^ [19,21]. Therefore, we could not specify clear definitions for large or small prostates. However generally speaking, our results in this systematic review and meta-analysis are more likely to apply when the prostate is smaller than 40 cm^3^ or larger than 100 cm^3^. Prostate specimen weight was used as a reference for the size in the majority of the included studies; however some studies reported on the size based on radiological estimation either by MRI or trans-rectal ultrasound (TRUS). Both MRI and TRUS are acceptable; however, MRI is slightly more accurate [34]. In addition, TRUS is operator-dependent [35].

In most of the included studies, operative time was defined as the time from starting skin incision until closure of the skin [9]. In fact, there are some steps, regardless of the technique, that are not affected by prostate size. For example, in robotic and laparoscopic surgery, insertion of the ports, robotic docking, and specimen retrieval are not affected by the size of the prostate. The same may be said for laparotomy incision and closure in open surgery. Assessing the time of prostate dissection separately could be more reflective of the impact of prostate size on operative time. In this meta-analysis, there was a trend towards shorter time for smaller prostates; however, comparing console time alone showed no difference. This could be explained by the smaller number of studies included in the comparison. In addition, many other factors could affect the operative time, such as the learning curves for both the surgeons and the assistants, and the performance of auxiliary steps such as nerve sparing and pelvic lymph node dissection [36].

It is well-known that organ hypertrophy is associated with angiogenesis and development of new vascularization [37]. Therefore, a larger prostate is expected to gain more accessory vasculature that might increase the chance of intraoperative bleeding. In our analysis, the blood loss had a significant and direct correlation with prostate size, but it did not show an impact on blood transfusion rates. However, this result can be useful in counseling patients with larger prostates and a higher risk of cardiac events in whom mild blood loss can induce clinical symptoms.

Surgical complications can be intraoperative, or either early or late postoperative. All of the included studies in this comparison displayed a higher incidence of complications with larger prostates; however, the controversy was about the significance of this difference. In the pooled analysis, larger prostates were associated with a significantly higher incidence of complications. This could be explained by the slightly longer operative time and the increased blood loss. However, there are other factors unrelated to prostate size that could affect the results, such as surgeon experience and patient comorbidities. Bladder neck stenosis was the only complication that could be assessed separately, but the comparison was very limited as only two studies were involved.

Functional outcomes of RP and postoperative quality of life are mainly based on postoperative continence and erectile function. Preservation of erectile function requires nerve sparing, which is not suitable for all patients. In patients with a Gleason score >7 or PSA > 20 ng/dl indicating high risk of disease recurrence, nerve sparing can increase the risk of PSM due to the close proximity of the neurovascular bundle to the prostate [30]. In our analysis, we focused on continence only, as there was not enough data to employ a pooled analysis for erectile function. Our comparison showed a trend towards better early continence with a smaller prostate. This could be explained by the smaller size of the bladder neck and the less traumatic effect of the apical dissection on the external urinary sphincter. However, there were discrepancies among the studies in the definition of continence and the assessment tools such as questionnaires.

The impact of prostate size on PSM rates is one of the most debated issues among the published reports. Moreover, it was the largest focal point of analysis in terms of the number of patients and studies included. We found a significantly higher risk of PSM linked to the smaller prostate. In a large prostate, the localized cancerous lesions might be surrounded by a thick benign tissue that acts as a barrier during dissection and prevents the breaching of the tumor. In a smaller prostate, the risk of breaching the tumor could be higher due to the thin layer between the tumor and the dissection plane. This might explain our findings. Smaller prostates also could be associated with a higher incidence of extracapsular extension of the tumor before surgery due to the previous theory; therefore, the higher incidence of PSM is due to a higher T stage. Ideally, comparison of the PSM should be stratified with cT stage and biopsy Gleason score; however, the reported data were not enough to investigate that. In concordance with PSM results, there was a trend towards a higher incidence of BCR in patients with smaller prostates. However, inability to reach the significance level could be due to the small number of included studies.

Despite addressing important questions, the results of this systematic review and meta-analysis should be carefully interpreted in view of major limiting factors: the retrospective nature of many of the included studies; the application of different protocols for the monitoring of surgical outcomes and continence recovery; the different surgical techniques and experiences among surgeons; the absence of a clear definition of large prostate; the variation in size assessment tools, either by using preoperative imaging or post-operative specimen weight; and inadequate consideration for other anatomical factors such as the dimensions of the true pelvis. All of these limitations should be considered in future studies in the interest of reaching a consensus regarding the impact of prostate size on RP outcome.

## 5. Conclusions

Based on our findings, the larger the prostate size, the higher the risk of blood loss and perioperative complications. However, the oncological outcomes are better compared to those in patients with smaller prostates. The impact of prostate size on functional outcomes is not clear. Well-designed prospective trials are necessary to investigate the effect of prostate size on radical prostatectomy outcomes.

## Figures and Tables

**Table 1:** Summary of the included studies.

Study	Type	Country	Technique	Size Measurement	Time Frame	Number of Patients
Chang 2005 [10]	Retrospective	UK	LRP	HPE	2000–2004	400
Freedland 2005 [28]	Retrospective	USA	Open	HPE	1988–2003	1602
Judd 2007 [9]	Retrospective	USA, UK	RARP	HPE	2004–2005	355
Adam 2008 [8]	Retrospective	USA	LRP	HPE	2001–2007	720
link 2008 [24]	Retrospective	USA	RARP	HPE	2003–2007	1847
Dubbelman 2009 [15]	RCT	Netherlands	Open	NA	NA	70
Finely 2009 [22]	Retrospective	USA	RARP	NA	NA	115
Joseph 2009 [23]	Retrospective	USA	Open+LRP	MRI	1998–2007	3067
Marteniz 2009 [16]	Retrospective	Canada	RARP	HPE	2005–2008	150
Tan 2009 [25]	Retrospective	USA	RARP	NA	2005–2009	1900
Sun 2012 [19]	Retrospective	Korea	Open	HPE	1993–2009	830
Si 2013 [18]	Retrospective	China	LRP	NA	NA	170
Takahiro 2014 [20]	Retrospective	Japan	RARP	HPE	2011–2013	219
Yosuke 2016 [26]	Retrospective	Japan	RARP	Ultrasound	2006–2013	436
Anastasios 2018 [14]	RCT	Italy	RARP	NA	2011–2014	79
Galfano 2018 [11]	Retrospective	Italy	RARP	HPE	2010–2015	750
Dong 2019 [27]	Retrospective	China	LRP	HPE	2002–2014	165
Regis 2021 [17]	RCT	Spain	RARP	MRI	NA	40
Thilo 2021 [21]	Retrospective	Germany	Open+RARP	HPE	2013–2018	929

RCT: randomized controlled trial, LRP: laparoscopic radical prostatectomy, RARP: robot-assisted radical prostatectomy, HPE: histopathological examination, NA: not available.
